# Cardiovascular and Cerebrovascular Implications of Growth Restriction: Mechanisms and Potential Treatments

**DOI:** 10.3390/ijms22147555

**Published:** 2021-07-14

**Authors:** Charmaine R. Rock, Tegan A. White, Beth R. Piscopo, Amy E. Sutherland, Suzanne L. Miller, Emily J. Camm, Beth J. Allison

**Affiliations:** 1 The Ritchie Centre, Hudson Institute of Medical Research, Clayton 3168, Australia; crroc1@student.monash.edu (C.R.R.); tawhi3@student.monash.edu (T.A.W.); bpis0001@student.monash.edu (B.R.P.); amy.sutherland@hudson.org.au (A.E.S.); suzie.miller@hudson.org.au (S.L.M.); emily.camm@hudson.org.au (E.J.C.); 2 Department of Obstetrics and Gynaecology, Monash University, Clayton 3168, Australia

**Keywords:** fetal growth restriction, brain sparing, cardiovascular, cerebrovascular, developmental programming, brain injury

## Abstract

Fetal growth restriction (FGR) is a common complication of pregnancy, resulting in a fetus that fails to reach its genetically determined growth potential. Whilst the fetal cardiovascular response to acute hypoxia is well established, the fetal defence to chronic hypoxia is not well understood due to experiment constraints. Growth restriction results primarily from reduced oxygen and nutrient supply to the developing fetus, resulting in chronic hypoxia. The fetus adapts to chronic hypoxia by redistributing cardiac output via brain sparing in an attempt to preserve function in the developing brain. This review highlights the impact of brain sparing on the developing fetal cardiovascular and cerebrovascular systems, as well as emerging long-term effects in offspring that were growth restricted at birth. Here, we explore the pathogenesis associated with brain sparing within the cerebrovascular system. An increased understanding of the mechanistic pathways will be critical to preventing neuropathological outcomes, including motor dysfunction such as cerebral palsy, or behaviour dysfunctions including autism and attention-deficit/hyperactivity disorder (ADHD).

## 1. Introduction

**Figure 1:**
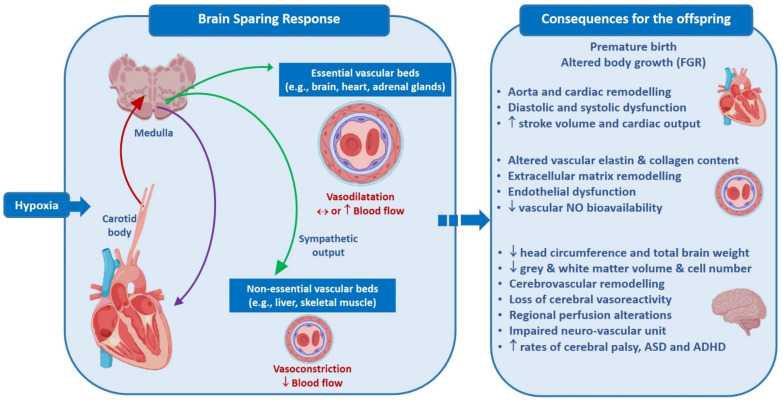
Brain sparing and consequences for the offspring. The fetus responds to hypoxia via afferent signals (red arrow) by redistributing blood flow to essential vascular beds (via efferent output, green arrows), a process known as brain sparing. Vagal control (purple arrow) decreases heart rate. This leads to a number of consequences in the fetus, particularly in the cardiovascular and cerebrovascular systems. Abbrev: autism spectrum disorder, ASD; attention deficit disorder (ADHD).

Suboptimal in utero growth is now well understood to have long-lasting physiological implications for the developing fetus and the subsequent newborn and adult. This narrative review will focus on the impacts of fetal growth restriction (FGR) on the cardiovascular and cerebrovascular system. One of the earliest physiological responses the fetus has to hypoxic stress is brain sparing. Brain sparing is an innate response that aims to preserve the development of vital organs, including the brain. Despite the nomenclature, brain sparing does not save the brain, and our group, amongst others, have shown subsequent brain [1], cardiovascular [2], and renal [3,4] injury. Indeed, it is likely that many of the mechanisms enacted throughout brain sparing, including chronic mal-activation of the autonomic nervous system and endocrine systems, contribute to the developmental programming of disease. This review examines the mechanisms underpinning the cardiovascular and cerebrovascular dysfunction associated with fetal growth restriction and brain sparing (Figure 1).

## 2. Fetal Growth Restriction

FGR is a common complication of pregnancy where the fetus fails to reach its genetically determined growth potential [5]. Depending on the specific definition of FGR, the incidence is up to 9% of pregnancies in high-income countries, and as high as 30% in low-income countries, with up to 30 million cases of FGR being diagnosed worldwide annually [6,7]. A consensus definition for FGR was reached in 2016 to include biometric measures of poor growth and functional parameters of placental dysfunction [8] and is summarised in Table 1. Prior to this definition, FGR and small for gestational age (SGA) were virtually used interchangeably. SGA refers to infants that are born with a birthweight less than the 10th percentile for their gestational age likely due to being genetically small, while an infant defined as FGR indicates pathological changes during pregnancy that contribute to suboptimal growth. FGR is linked to a myriad of adverse impacts including perinatal mortality, preterm birth [9] as well as pathology in the offspring which persists throughout childhood development and further into adulthood [10].

There are a range of fetal, maternal and placental factors that cause FGR. However, the most common cause is placental insufficiency, characterised by suboptimal placental function and abnormalities in placental blood flow, resulting in compromised oxygen and nutrient transfer to the fetus [11]. Optimal placental function and uteroplacental blood flow are essential for the maintenance of an adequate supply of oxygen and nutrients to support fetal development and growth. Any impairment in this supply can result in fetal hypoglycaemia and hypoxaemia [12]. Our understanding of FGR had been obtained from both human and pre-clinical animal models. The benefits and limitations of pre-clinical animal models of FGR have previously been reviewed [13].

## 3. Fetal Cardiovascular Response to Acute Hypoxia

The fetal cardiovascular system detects acute hypoxaemia and responds by redistributing its cardiac output towards essential vascular beds, such as the brain, heart and adrenal glands, at the expense of non-essential vascular beds (i.e., liver, skeletal muscle). This response is commonly referred to as *brain sparing* [9,13,14] and is the fetus’ attempt to minimise energy use [13]. Chemoreceptors located in the carotid body at the carotid artery’s bifurcation detect hypoxaemia, increasing sympathetic output to peripheral blood vessels via the fetal brain stem [14,15]. This increase in sympathetic output results in vasoconstriction in peripheral blood vessels [16].

Peripheral vasoconstriction initially occurs due to autonomic stimuli and is maintained by neutrally-induced increases in circulating fetal hormones [17,18,19]. Both the hypothalamic–pituitary–adrenal (HPA) axis and the adrenomedullary/sympathetic nervous system are employed to respond to stressors such as hypoxia. However, the efficacy of these systems matures over the course of gestation. Activation of the HPA axis in response to hypoxia results in the rapid release of corticotropin-releasing hormone and arginine vasopressin from the hypothalamus, which triggers adrenocorticotropic hormone release from the anterior pituitary and subsequent glucocorticoid production in the fetal adrenal cortex. The activation of the HPA axis in pre-clinical models of fetal hypoxaemia is demonstrated by a significant increase in cortisol, a key glucocorticoid [20,21]. Activation of the autonomic nervous system also causes release of vasoactive agents such as noradrenaline and other constrictor agents which maintain peripheral vasoconstriction [18]. Simultaneously, the parasympathetic response decreases heart rate and fetal breathing movements [22], which assists in minimising fetal oxygen consumption and energy expenditure [16]. Control of local vascular tone, which will be discussed in more detail, is crucial in the response to hypoxia in utero.

## 4. Fetal Cardiovascular Response to Chronic Hypoxia

Whilst the fetal cardiovascular response to acute hypoxia is well defined (see review [14]), the ability to study the fetal defence to chronic hypoxia is challenging. The development of large hypoxic chambers, which can maintain chronic severe hypoxia in sheep, has represented a great step forward in our ability to study the fetal defence to hypoxia. Allison and colleagues [23] utilised these hypoxic chambers to continuously monitor cardiovascular physiology during fetal hypoxaemia (PaO_2_~11 mmHg). This first description of the long-term fetal cardiovascular adaptations to chronic hypoxaemia, demonstrated that delivery of oxygen and glucose was increased to carotid arteries compared to femoral blood flow in hypoxaemic fetuses [23]. In addition, chronically hypoxaemic fetal sheep were significantly hypotensive for the duration of hypoxic insult, but were hypertensive by 1 year of age, which is comparable to adolescence in humans [24].

Ultimately, the cardiovascular adaptations to both acute and chronic hypoxaemia aim to slow fetal body growth, particularly in non-essential organs, which in turn results in asymmetrical growth restriction, clinically observed as an increase in head size relative to the body [14]. Importantly, while brain sparing is a physiological response aimed at maintaining blood flow and oxygen supply to essential organs in the face of an adverse intrauterine environment, this mechanism does not ensure normal fetal development.

To date, available data suggest that chronic hypoxaemia in utero produces key physiological adaptations including (1) autonomic nervous system stimulation, (2) altered circulating hormones [19,25], (3) oxidative stress, and (4) functional and morphological alterations to blood vessels [24]. Long-term exposure to hypoxaemia may permanently change the structure and function of the body, resulting in cardiac remodelling and dysfunction, increasing the life-long risk of cardiovascular disease. This review highlights the impact of these physiological adaptations to the developing fetal cardiovascular and cerebrovascular systems, as well as emerging long-term effects in offspring that were growth restricted at birth.

## 5. Cardiovascular Disorders and FGR

It is now well accepted that the foundations for cardiovascular health and dysfunction are laid down in utero, and being born growth restricted increases the predisposition to cardiovascular disease in adulthood. Numerous studies have examined the relationship between a poor uterine environment and the development of cardiovascular and cerebrovascular diseases in adulthood. Leon and colleagues [26] conducted an epidemiological study of ~15,000 births in Sweden between 1915 and 1929, and described a significant association between low birth weight and death from ischaemic heart disease in men aged 65 years and older following adjustment for socioeconomic circumstances. A cohort of 50-year-old males and females from Sheffield, UK showed an increase in systolic blood pressure of 2.7 mmHg for every ~450 g decrement in birth weight [27]. This inverse relationship remained significant after adjusting for sex, body mass index, alcohol intake and gestational age [27]. An Australian study examined the impact of birth weight and subsequent adult waist circumference in indigenous Australians born between 1992 and 1998. In this population, the lowest birth weight quartile had twice the cardiovascular disease risk compared to the highest birth weight quartile [28]. It is also now apparent that sub-clinical or cardiovascular dysfunction is present in FGR offspring early in neonatal life and adolescence, well before the onset of significant cardiovascular disease in adulthood.

## 6. Impact of FGR on Cardiac Function

Term-born SGA infants assessed via two-dimensional echocardiography 2–5 days after birth had diastolic dysfunction (reduced E and A wave velocities) and lower left ventricular output [29]. In a separate cohort of growth restricted infants born preterm, there was an increase in the thickness of the intima–media layers of the aorta as early as two weeks after birth [30]. Cruz-Lemini et al. [31] also found an increase in aorta intima–media thickness as well as cardiac septal thickness in SGA fetuses at ~37 weeks gestation compared to age matched appropriately grown (AG) fetuses. A follow-up study was conducted at 6 months of age where the increased aortic and cardiac septal thickness persisted in infants born small [31].

In a prospective study of 150 infants, Crispi and colleagues [32] compared cardiovascular morphology at 3–6 years of age in infants defined as either SGA or FGR with a control group of appropriate for gestational age infants. FGR was defined as infants with a birthweight below the 3rd percentile or abnormal cerebroplacental ratio on Doppler, while SGA was defined as an infant born between the 3rd and 10th percentile with normal cerebrovascular flow. Being born either SGA or FGR at birth altered cardiac morphology, resulting in globular-shaped hearts with increased stroke volume, cardiac output and left ventricular thickening. Both FGR and SGA infants had increased carotid artery thickness and increased blood pressure by 3–6 years of age, with a trend for the FGR infants having a higher blood pressure compared to SGA infants.

## 7. Co-Morbidity of FGR and Prematurity

FGR infants are frequently born preterm, and both FGR and prematurity compromise cardiovascular development, which makes it difficult to delineate the relative contribution of these factors on future cardiovascular function. Morsing et al. (2014) investigated a cohort of 7-year-old children born very preterm (27 ± 1 week) with FGR, and compared these infants to appropriately grown children born both at a comparable gestation or at term [33]. Premature birth, irrespective of birth weight, was associated with significantly increased blood pressure at 7 years of age. Interestingly, being born with co-morbidities of prematurity and FGR reduced aortic stiffness, increased distensibility coefficient, and impaired the microvascular response in the forearm to acetylcholine compared to the preterm appropriately grown infants [33]. Brodszki et al. [34] conducted a long-term prospective study comparing adolescents at 18 years of age who were born growth restricted in utero to appropriately grown subjects. In this cohort, FGR was associated with impaired blood vessel growth in central (abdominal aorta and carotid artery) and peripheral (popliteal artery) vascular beds relative to body size as well as a higher resting heart rate [34]. These data suggest that FGR can independently alter cardiovascular health and exacerbates the cardiovascular dysfunction associated with preterm birth.

## 8. Extracellular Matrix Proteins: Elastin and Collagen in FGR

The extracellular matrix proteins elastin and collagen are essential components of blood vessels. Elastin contributes to blood vessel elasticity and plays a role regulating cell proliferation and cell phenotype [35]. Adequate elastin is vital to allow reversible extensibility during the cardiac cycle. Conversely, collagen is a protein that is stiffer than elastin and provides structure to blood vessels. The relative stiffness of blood vessels is critical to cardiovascular health, with increased blood vessel stiffness being a recognised contributor to cardiovascular disease [36]. Blood vessel stiffness has been theorised to occur due to a transfer from elastin to collagen fibres [36]. It therefore follows that defects in elastin and or collagen fibre content or composition are frequently observed in adults with cardiovascular disease such as hypertension [35].

In humans, the deposition of elastin and collagen occurs rapidly in the last trimester of gestation and into the perinatal period [37]. The ratio of elastin to collagen is not fixed across blood vessels, with arteries nearer to the heart that experience higher blood flow showing increased elastic compliance, thus, an increased elastin to collagen ratio [38]. Flow through a blood vessel influences the ratio of elastin to collagen, and thus, pathological exposure to altered blood flow, such as during brain sparing in FGR, can alter the fine balance between these two proteins, and therefore, negatively impact blood vessel structure and function. Pre-clinical models allow further understanding of the impact of growth restriction on blood vessel morphology. In an ovine model of FGR, induced by umbilicoplacental embolisation, aortic remodelling was evident via reduced elastin content along with increased collagen deposition, resulting in media thickening and increased vessel stiffness [39]. In contrast, in an ovine hyperthermic model of FGR, the carotid artery [40] and thoracic aorta [41] showed increased collagen and elastin content and overall vessel stiffness. Studies in human infants have also demonstrated alterations to the extracellular matrix, with FGR infants demonstrating increased aortic collagen content, resulting in increased arterial stiffness, decreased arterial compliance and increased diameter change throughout the cardiac cycle [42], as evidenced by altered Doppler waveforms [30,43].

## 9. Vascular Reactivity and FGR

The altered structure of blood vessels, including the changes to the extracellular matrix, ultimately impact vascular function, which can be assessed by examining vascular reactivity. Examining vascular reactivity in a single vessel in isolation is unlikely to provide the full picture of the extent of FGR-induced vascular remodelling, given the chronically hypoxic fetus is undergoing redistribution of its cardiac output, with different vascular beds experiencing unique alterations to both flow and pressure. As vascular reactivity is best assessed in vitro, much of our understanding of vascular reactivity has been completed in pre-clinical models. One pre-clinical model of FGR, induced via umbilical artery occlusion in guinea pigs, assessed vascular reactivity in both the carotid and femoral arteries at the end of gestation and as the animals aged [44]. Paz and colleagues found impaired vascular reactivity in femoral, but not carotid arteries, evidenced by an increase in contractibility and deficits in nitric oxide (NO)-induced vasodilation [44]. With age, however, both carotid and femoral vessels had impaired NO-mediated responses. The authors suggested that although it is accepted that vascular NO-mediated responses decline with aging, exposure to placental insufficiency in utero accelerated the decline in NO-induced vasodilation, particularly in peripheral vessels within the guinea pig [44]. Adult baboon offspring born growth restricted following maternal nutrient restriction, were also found to have regional differences in the composition of vascular extracellular matrix proteins. Blood vessels in the periphery, such as the femoral and iliac arteries, had reduced vascular size and distensibility compared to central blood vessels, such as the carotid and brachial arteries, even after adjustment for body surface area [45].

Combined, functional data from human and in vitro data from pre-clinical models of FGR suggest that changes in the vasculature occur in concert with cardiac dysfunction. There is currently little understanding as to whether these pathologies occur in isolation or whether one precedes the other.

## 10. Vascular Endothelium and FGR

The vascular endothelium, a monolayer of endothelial cells, constitutes the inner cell lining of arteries, veins and capillaries [46]. The endothelium plays an essential role in the regulation of vascular tone [47], is instrumental for the formation of blood vessels during development, and initiates an inflammatory response and blood clotting in periods of tissue damage [46]. As the interface between the underlying tissue of blood vessels and the bloodstream, the vascular endothelium is responsible for sensing and adapting to local changes in blood flow, such as shear stress, blood composition, as well as hormones released into the circulation [48]. In response to these stimuli, the vascular endothelium releases endothelium-derived factors, including NO and prostacyclin, to induce vascular relaxation via the adjoining smooth muscle cells in the tunica media [46].

NO plays a vital role in regulating vascular tone, including vasorelaxation [49]. A decrease in the production of NO-mediated relaxation is often termed endothelial dysfunction due to the endothelium being the one of the key sites of NO production [49,50]. Endothelial dysfunction represents a key early step in the development of hypertension and atherosclerosis [51]. Endothelial dysfunction has been reported in pre-clinical models of growth restriction, induced by a variety of techniques including chronic hypoxia [24], single umbilical artery ligation [52] and cord occlusion [44]. In pre-clinical models, endothelial dysfunction is also present in adult offspring born growth restricted [24,44].

## 11. Vascular Homeostasis

The cardiovascular system constantly adapts to maintain blood pressure, and therefore, delivery of blood to tissues and organs as required. This is achieved primarily through dilation and constriction of blood vessels [48], known as vascular tone, which continually redirects blood flow to match the needs of each organ [48]. Vascular tone is mediated by many vasoactive compounds, including vasodilators NO, prostacyclin and endothelium-derived hyperpolarising factor, as well as vasoconstrictors thromboxane and endothelin-1 [49]. In response to increased blood pressure or shear stress, local vasodilators are released from the endothelial cells, instructing the smooth muscle in the tunica media to relax. This, in turn, results in the dilation of the blood vessel. The autonomic nervous system is responsible for regulating arterial blood pressure and systemic vascular resistance via direct (e.g.., noradrenaline release) and indirect (stimulating release of circulating hormones) methods to elicit responses at target organs. It is important to note that the population and efficacy of the vasoactive agents differ between different vascular beds [46].

## 12. Nitric Oxide (NO), Oxidative Stress and FGR

NO is synthesised in the vasculature in response to increased shear stress, or the presence of signalling molecules, (such as acetylcholine), which bind to receptors on the endothelial surface. However, NO is vulnerable to modulation by small molecules, including reactive oxygen species (ROS) such as superoxide anion. Like NO, ROS are continuously being produced; the balance between these two molecules is critical for maintaining vascular tone and thus regulating blood flow [14]. Several studies have demonstrated that modulation of the balance between ROS and NO, can alter the tone of the blood vessel and therefore blood vessel function [53,54]. Placental insufficiency and subsequent fetal hypoxaemia, is associated with increased ROS production and reduced NO bioavailability [24,55]. Both of these changes decrease the abundance of oxygen available for normal cellular functions, such as oxidative phosphorylation and the production of ATP [56]. The primary source of ROS in fetal hypoxaemia is likely to be the mitochondria, where molecular oxygen is unable to accept electrons at terminal complex IV, resulting in leakage of electrons and generation of the potent ROS, superoxide anion [57]. Increased superoxide anions rapidly bind to NO, directly scavenging and inactivating NO to form peroxynitrite [58]. Increased ROS can also impact NO bioavailability by suppressing the expression and function of endothelial nitric oxide synthase (eNOS) [59]. Both pre-clinical and clinical studies have shown reduced NO bioavailability in fetuses [2,60], children [61] and adult offspring [24,62] of compromised pregnancies [2]. A study in growth restricted rats showed that nitrotyrosine, a marker of peroxynitrite formation, is absent in control pregnancies but is present in the uterus of growth restricted fetuses [63].

In summary, cardiovascular consequences of a growth restricted pregnancy are evident from late gestation and have been shown to persist through to adulthood. The cardiovascular implications of growth restriction pervade throughout the cardiovascular system impacting molecular, structural as well as functional outcomes in the offspring. It is important to note that the cardiovascular system does not operate in isolation, but is incorporated into all other systems of the body through the blood vessels. The following sections will highlight how the control of blood vessels in the brain is impacted by growth restriction in utero.

## 13. Cerebrovascular Changes in FGR

As the name suggests, the cerebrovasculature is the component of the vascular system providing blood supply to the neuronal and glial cell populations of the brain. When under homeostatic conditions, comparable to that of systemic vasculature, cerebral blood vessels dynamically alter their vascular tone to match the metabolic needs of the local neural and glial cell populations [64]. A critical component of the brain sparing response is the vasodilation of the cerebral vasculature, which aims to protect and maintain brain growth [7]. However, recent research has highlighted the propensity for neurodevelopmental dysfunction following brain sparing associated with FGR [9]. Increasingly, pre-clinical and clinical research demonstrates that prolonged brain sparing may lead to cerebrovascular remodeling, loss of cerebral vasoreactivity, and increase the risk for neurodevelopmental dysfunction [2]. Understanding the pathogenesis associated with brain sparing within the cerebrovascular system is critical in preventing neuropathological outcomes, including motor dysfunction such as cerebral palsy, or behaviour dysfunctions including autism spectrum disorder (ASD) and attention-deficit/hyperactivity disorder (ADHD).

## 14. Detection of Brain Sparing In Vivo

Brain sparing can be detected clinically using Doppler ultrasound to visualise flow through the middle cerebral artery (MCA) and as such our evidence for the changes in blood flow distribution occurring with FGR is predominantly in human fetuses. Downstream vascular resistance can be determined by calculating the pulsatility index. A decreased pulsatility index within the MCA indicates blood vessel dilation and hence indicates brain sparing [65]. However, research investigating the patterns of redistribution of blood flow suggests that dilation of the MCA is evidence of an advanced stage of brain sparing [7,66], which is linked to worsening fetal hypoxaemia and poor postnatal outcomes including brain injury [67,68]. Although the MCA is the most common avenue for investigation of cerebral blood flow redistribution, it is not the first area of the brain that shows evidence of vasodilation associated with brain sparing [66]. Initially, in human fetuses, a decreased pulsatility index is observed in the anterior cerebral artery, demonstrating preferential supply of blood flow to frontal brain regions [66]. An exacerbation of chronic hypoxia then causes progressive dilation of the MCA, thereby shifting perfusion towards the basal ganglia [7], representing a group of subcortical nuclei responsible primarily for motor control, learning, behaviours and emotions. Combined, this evidence suggests that as FGR and brain sparing become more severe, region-specific alterations in cerebrovascular vasodilation and blood flow become evident.

## 15. Cerebrovascular circulation and the neurovascular unit in FGR

A robust and well-organised cerebrovascular network provides barrier functioning at the blood–brain interface, and comprises a symbiotic relationship between cells of the neurovascular unit (NVU) to moderate function. The NVU describes the close relationship between cells of the brain which together are fundamental for the control of cerebral blood flow, maintenance of the blood–brain barrier and cerebral homeostasis [69]. The NVU includes endothelial cells and pericytes, astrocytes, oligodendrocytes and microglia, as well as neurons [70]. The mechanisms underpinning the initial brain sparing response and associated molecular and functional changes occurring in the cerebrovasculature circulation and the NVU remain unknown. Pre-clinical models have enabled us to investigate the impact of FGR on the cerebrovasculature over the course of gestation.

A growing body of evidence from pre-clinical studies suggests that FGR has detrimental effects on the development of the cerebrovasculature and NVU components [71], which may explain, in part, why brain sparing is associated with neurodevelopmental abnormalities in infants [7,9]. An acute period of hypoxia (6–12 h) performed at mid-gestation in an experimental model of FGR in fetal sheep increased cerebral vessel proliferation and dilation in the white matter of the cerebral cortex when examined ~30 days after insult [72]. In contrast, long-term hypoxia associated with FGR, induced by single umbilical artery ligation in fetal sheep, has identified reductions in angiogenesis and vascular density within the white matter 24 h after birth [9]. The effects of FGR induced by exposing sheep to high altitude in pregnancy have further highlighted changes in the size, shape and function of cerebral blood vessels, including increased wall thickness and reduction in vascular contractility in late gestation fetal sheep following 110 days hypoxia [73]. It has been hypothesised that these changes in cerebrovasculature in FGR neonates are likely due to the downregulation of the angiogenic factor vascular endothelial growth factor (VEGF), or a reduction in the proliferation of endothelial cells [74,75]. This variability may reflect a temporal and spatial variation in the activation of angiogenic factors during FGR or the timing and duration of hypoxic insult.

In addition to a reduction in vessel density within cerebral white matter, FGR induced via single umbilical artery ligation in fetal sheep also leads to reductions in pericyte and astrocyte end-feet coverage of blood vessels [9,76]. Pericytes are critical to the maintenance of the blood–brain barrier but also play an important role in angiogenesis including the initiation of new growth and maturation of existing blood vessels [77]. Astrocytes are vital to vascular integrity, hence a reduction in astrocyte connections with blood vessels will likely lead to an overall weakening of the structure of the vasculature [9]. The combined impact of both pericyte and astrocyte loss in the cerebrovasculature of FGR neonates will ultimately impact cerebral perfusion and vascular contractility, whilst also increasing the risk of haemorrhage or hyperdilation of blood vessels [76,78]. Further research is required to elucidate how FGR acts to alter the developing cerebrovasculature, and in turn, impacts the cells of the NVU. Our understanding of the effects of altered NVU structure on long-term functional neurodevelopmental outcomes in growth restricted infants remains unknown.

## 16. FGR and Neurodevelopment

An extensive body of research shows that an adverse intrauterine environment is a key factor underlying altered brain development in the offspring [22]. While perinatal mortality and preterm birth are the most immediate consequences of FGR, neurodevelopmental deficits are also strongly associated with FGR and have sustained impacts throughout the individual’s lifespan [79,80]. The association between FGR and neurodevelopment deficits is well supported across an array of clinical and experimental studies, with both structural and functional outcomes linked to suboptimal fetal growth [7,9,79,81,82,83].

## 17. Long-Term Neurological Implications of Brain Sparing

Imaging studies using magnetic resonance imaging (MRI) show that FGR in human infants is associated with reduced head circumference [84], and decreases in total brain weight, grey and white matter volumes, and total cell number [85,86,87]. MRI studies have also demonstrated changes in structural brain networks in FGR neonates [88]. Taken together, these results suggest that brain sparing does not necessarily preserve normal brain structure [3,85,89].

Haemodynamic changes in the fetal cerebrovasculature and the NVU have been linked to neurobehavioural outcomes in the offspring. Deficits in areas such as communication and personal–social interactions have been reported in SGA infants at 2 years of age who demonstrated abnormal fetal MCA Doppler ultrasounds [90]. Further, emotion and attention issues have been observed at 18 months of age in infants born FGR with demonstrated dilation of the anterior cerebral artery at preterm gestational age (median age 30 weeks) [79]. Although it is clear that brain sparing occurs as an adaptive cardiovascular response to chronic hypoxia, it does not appear to spare the brain from cerebrovascular remodelling nor adverse neurobehavioural outcomes in the offspring.

Structural deficits in the brain of growth restricted infants are likely to underlie functional deficits observed in children with FGR. Functional deficits are broad in how they manifest, but may include significant impairments in motor function including cerebral palsy. Term-born children who were born extremely growth restricted may be less cognitively equipped for school, have difficulty with problem solving tasks, and be more impulsive compared to their appropriately grown peers [82]. A retrospective cohort study of 4503 singleton children born at term or near term with a birthweight below the 10th percentile (SGA) were at a 4–6 fold increased risk of having cerebral palsy [91]. A 10 year prospective study of 123 term-born children showed that infants who experienced brain sparing as assessed by ultrasound during pregnancy were found to have a significantly lower IQ than the appropriately grown controls by ages 9 to 10 [92]. However, it is important to note that the prospective study by Leitner and colleagues was not adjusted for any confounding factors such as socioeconomic factors or educational background. Whilst many research papers have focused on the association between FGR and cerebral palsy, there is also emerging evidence of a link between FGR and neurodevelopmental disorders such as ASD and ADHD [93,94]. There is a strong overlap in the brain regions that are highly vulnerable to injury with FGR and associated with ASD, such as the hippocampus and the cerebellum [7,95]. A similar relationship exists between FGR and ADHD, where both conditions are linked to deficits in attention and increased levels of hyperactivity [22,96]. Further, many of the pathways known to be linked to ADHD and ASD are similar to those linked to FGR including increased oxidative stress and poor autonomic control. There is a paucity of data regarding the potential link between ADHD and ASD and being born FGR. Our group is currently investigating the potential link in our ovine model of FGR induced by single umbilical artery ligation.

## 18. FGR and Neurodevelopmental Disorders

The pathogenesis of ADHD and ASD remains unknown. However, there is an emerging role for poor cerebral perfusion and/or control of cerebral blood flow [97,98]. The association between impaired cerebrovascular function and neurodevelopmental disorders is perhaps not surprising given the fact that neuronal activity is tightly coupled to blood flow. Rennie and colleagues [99] demonstrated impaired control of cerebral blood flow in a small number of low birth weight infants that subsequently were diagnosed as having poor neurodevelopment. Functional imaging of children with ADHD find regional hypo- and hyperperfusion within critical brain regions, implicating cerebrovascular alterations. Early imaging studies in children at a median age of 10 years with ADHD found reduced blood flow in regions involved in voluntary movement, and increased blood flow to regions associated with visual processing, with no change in overall blood flow [100]. More advanced imaging has shown discrete, region-specific changes in cerebral blood flow, with reduced flow in regions associated with decision making, attention deficits and hyperactivity, and increased blood flow to regions associated with movement [101]. In contrast to ADHD, ASD is associated with significant and region-specific reductions in blood flow, specifically in the bilateral frontal lobe, which controls judgement and reasoning [102,103]. Animal models of poor placental function have mirrored these clinical findings, and have shed light on the impact of poor prenatal growth on behavioural disorders and cerebral vasculature. For example, Kay and colleagues found that adult mice with placental growth factor deficiency (*Pgf*^−/−^) had altered cognitive function and an increased density of cerebral blood vessels [104]. Interestingly, the increase in cerebral blood vessels occurred due to a significant increase in small diameter vessels in *Pgf*^−/−^ mice [104]. Therefore, cardiovascular structure and function may be a potential driver linking FGR and neurobehavioural outcomes. However, it is important to note that the causes of ASD and ADHD remain under investigation, and the studies highlighted above indicate that changes in blood flow, and its neurovascular origins, may be contributory.

## 19. Conclusions

It is well accepted that brain sparing is a vital response to a prenatal insult/adverse prenatal environment, and acts as a protective mechanism to preserve brain development and confer survival of the developing fetus. Prolonged brain sparing and the subsequent response of the fetal cardiovascular system can program long-term cardiovascular dysfunction in the offspring. In addition, the persistent redistribution of cardiac output may impact the developing brain via alterations to the cerebrovascular circulation, contributing to long-term neurological deficits (Figure 1). A number of critical knowledge gaps remain, particularly around our understanding of the pathways and mechanisms involved in the mal/adaptive cardiovascular response to poor placental function, and the potential link between brain sparing, altered structure of the cerebrovasculature, and long-term neurological implications. Only with this understanding will we be able to move forward to examine therapeutic options for growth restricted fetuses that protect various organ systems.

## Figures and Tables

**Table 1:** Consensus definition of early and late onset FGR.

	Early FGR	Late FGR
**Gestation**	<32 weeks	>32 weeks
**Congenital anomalies**	absent	absent
**Estimated fetal weight or abdominal circumference**	<3rd percentile or absent end-diastolic flow in the umbilical artery	<3rd percentile
**OR**		**OR 2 out of**
**Estimated fetal weight or abdominal circumference**	<10th percentile	<10th percentile or crossing > 2 quartiles on growth percentiles
**Uterine artery pulsatility index**	>95th percentile	
**Umbilical artery pulsatility index**	>95th percentile	>95th percentile
**Cerebroplacental ratio**		<5th percentile
